# Supplementary material for: Are carbon emissions trading and green financial instruments synergistic? -Comprehensive quantitative research based on content analysis
Source: PLoS One. 2024 Mar 7;19(3):e0298601. doi: 10.1371/journal.pone.0298601 (PMC10919601; doi:10.1371/journal.pone.0298601)
Supplement: S1 Data — (ZIP) [file pone.0298601.s001.zip › Data/地级市Z_F工作报告.zip!/╝¬┴╓/╦─╞╜╩╨/└┤╫╘╣1⁄2╓┌║┼í╛┬φ┐╦╩2╛▌═°í┐_2012.docx]

各位代表：

　　现在，我代表市人民政府，向大会报告工作，请予审议。

　　一、2011年工作回顾

　　过去的一年，在省委、省政府和市委领导下，全市上下深入实施“一核三带”富民优先战略，强力推进“三保两调一努力”重点工作，攻坚克难，求富图强，呈现出经济发展阔步前行、财税收入攀升晋位、城建规划历史跨越、社会民生明显改善、干部群众精神面貌焕然一新的喜人局面，圆满完成《政府工作报告》确定的各项任务，实现了“十二五”强势开局的良好态势。

　　——综合经济实力赶超提升。预计全市地区生产总值近1000亿元，增长20%，其中三次产业增加值分别增长9%、30%和37%。固定资产投资完成550亿元（新口径），增长33%，其中工业投资增长60%以上。全口径财政收入连续跨越60亿、70亿两个关口，完成73.6亿元，增长31.4%；地方级财政收入跨越30亿关口，完成38.9亿元，增长39.4%；市本级地方级财政收入完成17.5亿元，增长44.6%，实现两年翻番，其总量由全省第八位跃升到第四位，在阳光津补贴大额支出的情况下，已把体内收支长期不平衡的帽子远远抛弃了！全市规模以上工业总产值、增加值、利润分别完成1460亿元、440亿元、50亿元，分别增长48.5%、25.6%和43%，工业经济总量前三季度跃升至全省第三位。社会消费品零售总额达到339亿元，增长17.8%，居民消费价格涨幅控制在4.8%以内。外贸出口额实现8200万美元，增长21%。奋力直追，在数对数的评比条件下，我市荣获全省“发展杯”竞赛活动第二名的业绩。

　　——经济发展格局初步形成。为把“一核三带”战略蓝图落到实处，邀请国内外知名规划设计机构，高标准编制城市总体规划、土地利用总体规划、东部生态新城规划和城市公交、供热、供水、公厕、市场、教育、医疗、消防等21项专项规划及水利、林业、环保等11项重大项目规划，中心城区控详规覆盖率达到60%，这是我市建市以来，第一次全覆盖、分专项、大尺度编制城市发展规划。围绕发展工业经济，编制完成中欧专用车园区、中韩新能源园区、两岸化工食品医药园区及地方与央企合作的平东老工业区四大特色园区规划。“一湖两区”旅游规划已经完成，“一核三带”节点镇发展规划开始编制。我市被确定为国家中小城市生态环境建设实验区、国家可持续发展实验区、国家振兴老工业基地重点城市、省先进装备制造业新型工业化示范基地、省专用车生产基地。军民合用机场筹建工作正式启动。梨树、铁东工业园区即将提升为省级开发区，省规划管理园区、特色园区发展迅速，数量居各市州之首。初步构筑起经济发展的产业与空间新布局，为我市中长期发展奠定了坚实基础。

　　——制约发展瓶颈有效破解。金融环境持续改善，区域内资金流量快速增长。13亿元城投债券发行到位，为城市基础设施与园区建设提供有力支撑。中小企业担保公司和小额贷款公司发展到36家，发放贷款26亿元；江西南昌银行、吉林九台农商行、吉林银行村镇银行分别落户铁东区、公主岭市和双辽市，省5家农村资金互助社全部落户我市，银企对接初见成效，畅通了中小企业及农村经济发展的金融血脉。资金存量不断增加，预计全市存款余额达到650亿元，增长20%。吉春药业公司完成股改，精细化工公司实施股权融资，上市步伐明显加快。市财政、发改、社保、交通、人社等部门共争取专项资金17.1亿元，增长30.3%；总部经济积极推进，市地税、国税、财政、畜牧等部门协调引入总部经济见到成效，市区新增税收1.2亿元。严格执行土地招拍挂制度，市本级收取土地出让金全价款10亿元；获准项目用地688.5公顷，是上年的1.2倍，也是多年来项目用地最多年份。争取国家农村土地综合整理项目4个，市区到位资金6000万元。双辽、梨树和公主岭工业集中区争取“以奖代补”资金总额2400万元。

　　——项目招商形成热潮。围绕“一烟二油三车四光伏”，全市建设3000万元以上项目875个，其中亿元以上252个，总投资超过1100亿元。“4.18”以烟厂易地扩能改造为代表的全市首批46个亿元以上项目集中开工，在全省率先拉开项目建设序幕。积极推进“南接北融”，坚持迈大步走出去、邀高端请进来，组织参加大型经贸交流活动，主动上门与部分央企、民企集团及国内外著名中介咨询机构开展项目对接和专题招商。利用“两会”等有利时机，访部委、走厅局、跑资金、盯项目。由市人大、政协等领导牵头的七个项目推进组工作全面启动。举办商务班集中培训35名干部，专业招商团队显露雏形。一些企业积极引进战略投资者，宁做大企业股东不做小老板，实现迅速扩张。新天龙并入吉粮集团，金士百加速推进股改，奋进改装车与中航集团开展合作，一汽专用车成功与法国飞马特集团合资，四平昊华易地扩能改造获得批准。一大批重点项目迅速落地，形成了你追我赶的喜人局面。

　　——现代农业转型升级。积极推进“三区一建”。加快发展粮油高产示范区、棚室蔬菜园区、牧业小区，分别新增30个、23个和356个，数量居全省前列。万亩蔬菜棚膜工程农业标准化国家示范区5个。铁东区以活动引领村镇环境建设效果明显。粮食产量再创历史新高，商品量突破150亿斤。农产品加工业销售收入397亿元，增长32%。公主岭、梨树进入全国产粮大县前5强，公主岭被命名为中国玉米之乡，成功举办中国玉米节和玉米产业博览会。双辽市被授予全国生猪生产大县称号。伊通县被评为全国首批农业产业化示范基地。以梨树县为代表的全市农民专业合作社发展到3287户，覆盖全市所有行政村，保持全省领先。国内首家粮食信托合作社在我市创建。省十年绿化美化吉林大地我市通过验收。县域经济取得新进展，县域生产总值同比增长21%，公主岭市进入全省县域综合发展前5强。四县三区不甘居后，勇闯发展新潮头。

　　——城乡建设实现跨越。全市城市基础设施投资完成73.5亿元，其中市区完成27亿元、实施53项重点工程，是历史上最多的一年。市区维修改造40条街路，路灯主体完成节能改造，群众关心的市区道路状况全面改善，主次街路面貌焕然一新。启动“中”字型快速路建设，交通拥堵现象得到有效缓解。高铁站前紫昕广场、旭日立交桥开工建设，规划馆工程进度加快，现代化东南新城正在施工建设。建设西湖水上公园，城市南部横向景观轴线全面启动，一个都市形态的四平城即将屹立起来！完成城区西南及辽源方向出口绿带建设，新增绿地92.2万平方米。打击抢种抢建等违法行为，提高了城市土地价值和利用效率。全市建设保障性住房247.3万平方米，完成暖房子工程改造150万平方米，农村泥草房改造7971户，中低收入群体住房条件明显改善。新建和改造燃气管网84公里、供热管网58公里、污水管网11公里，主要道路零标高以下良心工程高质量完成，为解决道路“拉链”问题打下基础。完成热电厂二期第一台机组建设及小锅炉撤并等工程，冬季供暖得到有效保障。广电大厦、影发大厦、电业宾馆等烂尾工程恢复重建，即将投入使用。伊通等4个县（市）和辽河区的5个新城建设全面铺开，市容市貌进一步改观，县城及小城镇建设取得新成果。

　　——民生事业富有成效。深入实施“工资倍增计划”，市区财政供养人口津补贴标准，由全省末位迅速上升到第四位。预计城镇居民人均可支配收入、农民人均纯收入实现17800元和7200元，分别增长8%和10%，让群众分享改革发展成果。新增就业6.4万人，再就业6064人。6个县（市）区全部纳入新农保和城居保国家试点，成为全省首批全覆盖市（州），60周岁以上老人基础养老金全部发放到位；厂办大集体职工和“五七家属工”参保续保工作基本结束；城镇职工、居民基本医疗保险全覆盖，初步实现人人享有基本社会保障。城市低保金月提高至275元，农村低保年人均增至1647元，实行 “双联动”，使困难群众生活随经济发展而提高。积极推进“社区行动计划”，社区服务功能不断完善，长征、拥军两个社区被评为全国文化先进社区。深入开展“三走进、三帮扶、三提升”活动。教育教学质量进一步提升，升学考试纪律全面加强，加固重建校舍89.3万平方米，开工率达到90%以上。实施文化惠民工程，建设乡镇综合文化站52个、农家文化大院69个。通过市场化运作，成功举办中国·四平汽车拉力锦标赛。我市荣获中国最具投资价值旅游城市称号，龙山湖被命名为国家级水利风景区。“创文明城市、做文明市民”活动不断深入。深化医疗卫生体制改革，115家基层医疗单位实现药品零差价销售，评选“十所优质医院、百名满意医生”活动受到社会关注和群众好评，工作经验在全国推广，城乡医疗服务能力不断增强，初步缓解了看病贵问题。民生实事的推进，使百姓受益，提升了党和政府的公信力。

　　——政府建设改善加强。始终在“四型”政府上下功夫，公务人员的经济意识切实强化，服务水平有所提高，管理方式明显改善，廉洁自律得到加强，各级政府狠抓落实，提高执行力。坚持定期向市委报告工作制度，认真执行人大及其常委会决议，积极开展政治协商，自觉接受人大法律监督、工作监督和政协民主监督。办理人大代表建议99件、政协委员提案188件，办复率100%。行政监察、审计监督不断加强，依法行政能力进一步提高。围绕调整机构编制，取消162个自定编制，清理退出事业单位28家，收回事业编制2469名，核销1131名，首次实现编制与人员“负增长”，“大家吃、吃大家”的惯性问题从此刹车。经济发展软环境由全省末位跃升至前列，双辽市软环境建设连续三年受到省政府表彰。每季度在媒体通报各县（市）、区主要经济指标及纳税百万元以上企业缴税情况，营造出你追我赶的发展氛围。治安防控和矛盾纠纷排查调处体系逐步完善，公安机关追逃“清网行动”受到省公安厅通令嘉奖。食品药品监管力度加大，安全生产形势基本稳定。开展“关爱老兵、情系老兵”帮扶活动，探索出了破解矛盾的新方法。新一轮全国双拥模范城检查我市通过验收。民族宗教、档案、地震、老龄、地方志、外事侨务等工作取得新进展。

　　过去一年，政府上下扎实推进“三保两调一努力”重点工作。“保财政收支”做到有效破解财力弱、负担重、底子薄的困境，真正达到体内平衡，实现财政收支状况的新转折；“保工业运行”做到积极发挥企业家作用，通过奋力抢拼，使在全省排名第四的工业经济总量，前三季度跃升到第三位，带来赶超晋位的新希望；“保城市建设”做到初步完成零标高以下良心工程，全方位编制规划，高水准开发改造，形成城市发展的新格局；“调机构编制”做到摸清底数，核销减少，理顺职责，严格控制增编进人，建立起规范管理的新机制；“调阳光政务”做到注重执行落实，着眼提高效率，为实现阳光政务做好基础准备，呈现出积极进取的新变化；“一努力”就是把民生实事办成办好，实施环境改观，养老、医疗保险新覆盖，社区建设重拳出击，政府为民服务工作又掀新热潮。

　　各位代表！过去的一年，是奋起赶超、合力攻坚的一年，更是破解难题、筑牢基础的一年。发展成绩的取得来之不易，主要得益于市委的核心作用和市人大、市政协的鼎力支持，得益于检法两院、党群部门、中省直单位的积极参与和大力配合，得益于广大干部群众的同舟共济和艰苦努力。在此，我代表四平市人民政府，向奋战在全市各条战线上的广大劳动者，向给予政府工作大力支持的人大代表、政协委员、各民主党派、工商联、人民团体和社会各界人士，向关心、支持、帮助四平建设的驻平部队、武警官兵及海内外朋友表示崇高敬意和衷心感谢！

　　回顾过去一年，我们也清醒认识到，全市经济社会发展仍然存在一些突出矛盾和问题。经济总量不大，科技含量不高，内生动力不足；工业支撑能力不强，服务业发展相对滞后；部分县（市）缺乏支柱产业和税源型龙头企业；财税增收空间有限，人均财政收支水平偏低；群众对城市管理和治安环境还有不满意的地方，不稳定因素还不同程度存在；公务人员市场意识需要强化，服务效率有待提高。我们要高度重视，采取措施，认真加以解决。

　　二、2012年工作任务

　　今年是完成“十二五”规划目标的承上启下之年。做好今年工作，要紧紧围绕发展和民生两大主题，认真贯彻市第六次党代会精神，深入实施“一核三带”富民优先战略，落实“四突破五确保”的工作任务,实现“六增力一改观”的奋斗目标，全面完成全市生产总值增长20%,工业总产值、固定资产投资和全口径、地方级财政收入增长30%以上，城镇居民人均可支配收入和农民人均纯收入增长10%以上。具体要做好四个突破、五个确保。四个突破是：

　　——在项目开发建设上实现突破。突出靠大联强和嫁接改造，依托开发区和四大特色产业园区，探寻新的市场空间，发挥各级干部和专业团队能动作用，突出领导责任和企业积极性，建立调度、问责、承诺等制度保障，对项目报批、征地、开工、推进、投产、达效等全过程跟踪服务，实现建设10亿元以上大项目的新突破。

　　——在要素优化配置上实现突破。企业是市场主体，各级政府要为企业发展提供资金、技术、人才、土地等要素支持。积极争取各方面资金，创新土地开发利用模式，千方百计招商引资，引进先进实用技术和高端人才，研究聚集资金、盘活资产和开发资源的有效措施，加速资源向资产、资产向资本转化进程，实现要素配置上的新突破。

　　——在城市建设管理上实现突破。把东南新城拓展作为重要目标，全力组织实施，拉开发展新格局，形成新的增长极。继续做好老城区疏理，推进零标高以下良心工程、零标高文明工程和零标高以上景观工程建设，改善人居和创业环境，实现城市整体增值，打造区域中心城市，实现人居环境建设上的新突破。

　　——在社会管理创新上实现突破。强化政府社会管理和公共服务职能，推进社会管理综合治理，完善城市管理，改善治安环境，形成司法调解机制，强化社区长效管理，落实信访积案包保责任，提高安全意识，落实监管责任，认真做好应急预案，妥善处置突发事件，推进文化体制改革，丰富群众文化生活，实现创建稳定和谐四平的新突破。

　　四个突破是工作方向的延伸定位，五个确保是工作任务的具体展现。为此，要实现以下目标：

　　（一）激活发展动力，确保工业经济高速增长

　　加快企业转型升级。积极争取与中航集团、中联重科、海翼集团等央企、民企集团开展合作，实现嫁接改造。支持新天龙、金士百、巨元、天成、四平专用车等企业转型升级。加大企业技改力度，推动重大科技成果转化，新产品产值率超过10%。强化包保服务责任制，以4月18日为新起点，开工新建一批重大项目。

　　加快企业上市步伐。推动吉林世宝公司随母公司在A股上市、吉春药业公司在中小板上市。支持精细化工公司、天成玉米公司等11户企业做好上市培训。寻找战略合作者，寻求政策、信息和技术支持。各类专项资金向重点企业倾斜，争取奖励政策，激发企业热情，使更多企业进入资本市场。

　　推进重点产业发展。倾力打造换热器、整车及专用车、汽车零部件、光伏四大产业，形成支柱产业集群。加快专用车产能扩张、高性能车开发、零部件配套体系及专用车展销中心建设；开发高端节能换热器、光伏太阳能电池；化工产业向精深化、高端化、规模化方向发展。确立政策导向，筹集国有资本，全力向四大特色园区聚集，努力做好开发区及园区的三通一平等前期工作。各县（市）区、开发区都要确定1-2个支柱产业园区，形成区域优势产业集群。

　　积极做好要素保障。注重市场研究，强化营销意识，拓宽销售渠道，提升品牌效应，多元化挺进全国大市场。把净利润率作为衡量新上项目和评价企业的主要依据，加大支持扶持力度。继续放大13亿元城投债券，加强银企对接，发挥融资平台和创投基金作用，筹建吉林创业投资引导基金四平子基金，加速资本聚集流入。摸清改制后国有企业及机关事业单位资产，研究探索资产置换，落实土地收储制度，积极开展招商引资和向上争取资金。通过资金支持和优惠政策，吸引人才到园区建功立业，引导科技人才到孵化基地创业。

　　切实发挥企业主体作用。注意充分调动企业积极性，通过企业引进战略投资者，主动嫁接改造，实现增量扩张。为纳税人创造宽松的政策和优质的服务环境，统筹社会公共资源，给予突出贡献企业家政治及医疗、保健等方面的特殊待遇，赋予殊荣，厚爱一分，高看一眼，尊重财富创造者！

　　（二）强化政策和服务，确保“三经”大发展

　　加快发展民营经济。通过重点扶持，引进合作伙伴，培育规模民营企业，形成大中小梯次发展结构，为进入资本市场创造条件。继续推进全民创业，采取资本金政府补助、税收返还、融资担保、规费减免等措施，扶持硕博毕业生、文化创意人员、信息技术人员等人才来我市自主创业，扩张总量，发展智力型企业。学习重庆等地扶持小微企业发展经验，设立创业基金，建设中小企业孵化园，助推民营经济发展。

　　大力发展服务经济。立足我市优势特色，在发展商贸等传统服务行业同时，大力发展金融保险、服务外包、文化创意、数字动漫及社区服务等现代服务业。吸引直辖市及省会城市高端人才，引领创新创业，发展智库经济，打造“智慧四平”。推进红酒交易中心、专用车交易中心、大岭汽车物流园区、博来德现代物流中心建设，努力打造辐射东北四省区的商品集散地。发展以叶赫满族风情园区和叶赫生态博物馆为主体的旅游业。

　　全员动手发展总部经济。县（市）区，尤其市直部门要发挥作用，积极吸引企业、科研、教育、文化、广告、设计等各类集团机构来平设立办事机构或区域中心，发挥总部经济增加人流、物流、资金流及税收效应，实现产业聚集、人才进驻、资本流入的积极效果，形成区域经济发展核心竞争力。制定优惠政策，为域外企业总部入驻四平解决办公场所，设立扶持专项基金，开辟“绿色通道”，在工商注册、税务登记等方面特事特办，享受社会资源优惠待遇。投入专项资金，完善基础设施，创造总部经济发展的优越条件。

　　（三）突出发展县域经济，确保现代农业扎实推进

　　加快培育优势特色产业。进一步加大政策扶持力度，延伸产业链条，发展税源型企业，培育产业集群。积极转变农业发展方式，引进大企业集团，实施集约化经营，发展无公害、绿色、有机食品，打造农业名优品牌，各县（市）、区都要建立重点先导区。

　　加快传统产业向现代农业转变。强化“三区一建”示范带动作用，促进粮油高产示范区技术应用集成化，提高粮食高产稳产水平。推进棚室园区规模化，建设市场稳定、特色鲜明的东北蔬菜基地。推进标准化牧业小区建设，打造安全优质、全国知名的四平畜牧品牌。积极探索农民合作社、“三区一建”、土地流转、农村社会管理创新四项工作有机结合，争取在全国再树新典型。

　　加快涉农基础设施建设。坚持政府引导、农民主体、社会参与，推进“一核三带”节点镇连片治理，培育区域样板村群，全部完成村村通水泥（沥青）路任务，改善农村人居环境。抓住国家支持水利发展政策机遇，积极争取水利项目和资金，加快龙山湖引水二期工程建设，确保按期通水；扎实推进南北河治理，促进转山湖引水、各县（市）城市防洪和灌区节水改造等重点水利工程建设，把我市农业优势转化为经济优势。

　　（四）坚持疏理与拓展并举，确保城市建设管理实现新跨越

　　加快新城区拓展开发。强力推进东南生态新城建设，“八馆一中心”和公检法建筑群规划设计全面铺开，规划展览馆、科技馆早日建成投入使用，旭日立交桥、综合客运枢纽、人防指挥通讯中心按期竣工。积极推进各县（市）的新城建设，提升城镇化水平。

　　搞好老城区疏理改造。加快控制性详细规划编制，实现城区全覆盖。充分发挥规划委员会、专家和群众监督作用，确保严格执行规划。加快重点污染企业退城进区步伐，完成殡仪馆易地重建和烈士陵园改建规划，制定北山绿化美化实施方案。启动“两河四岸四湖”景观建设，开发城市生态水系。栽植多年生大树2万棵，人均公共绿地面积实现10平方米，逐步解决林疏问题。搞好树木、桥梁、广场及景观路两侧楼体亮化改造。投入16亿元，开工建设道路、桥梁等项目60项，建设公铁立交桥5座，城区主次街路全部改造完毕。加快推进快速路建设，建设简易立交桥和返回式环岛，进一步提高道路通行能力。继续推进四平军民合用机场项目建设。新增保障性住房46万平方米，完成暖房子工程200万平方米。结合人防工程，建设地下停车场。高标准改造仁兴商业步行街。加大土地、规划的执法监察力度，严厉打击违法用地和违章建筑。进一步理顺管理体制，推进数字化城市进程，突出整治牌匾及户外广告，下力量解决环境卫生、占道经营和小区管理等群众关心问题，让四平更美、更靓、更有人气和魅力！

　　（五）办好民生实事，确保居民生活品质显著提升

　　围绕解决群众关心的问题，把民生事业更加生活化、实效化、具体化，切实办好衣、食、住、行、乐及教育、医疗、就业、养老、和谐等10个方面重点实事。

　　衣，主要是解决商业网点布局问题，推进社区服务社会化，满足群众多样化消费需求。食，主要是解决食品药品安全问题，加强环境保护工作，加大监督检查力度，让群众吃得更安全更放心。住，主要是解决商品房和廉租房供应结构问题，优先保障无房户和困难户住房需求，继续改善中低收入群体住房条件。行，主要是解决通畅问题，优先发展城市公共交通，投入资金更新公交车150台,打造4条公交精品线路。乐，主要是解决活跃问题，积极推进“社区行动计划”第二阶段工作，继续开展丰富多彩的群众性文体娱乐活动，提升四平人的精气神。

　　教育，重点是坚持育人为本，师德为先，加强学风教风和学校环境建设，着力解决教育公平及安全问题。医疗，重点是扩大医保覆盖面，提高保障水平，打造放心医院，争做满意医生,突出解决看病难看病贵问题。就业，重点是通过发展经济，增加就业，努力做到居民收入与经济增长同步。养老，重点是通过保险、民政以及养老大院等多种形式，积极做到老有所养。和谐，重点是解决群众关心的热点难点问题，严厉打击绺窃、盗抢等违法犯罪活动，切实加强安全生产，积极应对突发事件，全力做好信访工作，确保社会安全稳定，让四平人民群众生活的更有质量、更有尊严！

　　各位代表！全面完成今年各项目标任务，对各级政府提出了更高的要求，必须继续加强政府自身建设，推进政府管理创新。

　　建设创新经济型政府，做到“懂经济、会算账、抓项目”。把行政行为与市场行为一致起来，在公共产品提供、招商引资上项目、企业培育、城市建设过程中，严格进行成本核算，以最合理的投入为人民办更多实事。建设懂经济、抓项目、会落实、能干事、守护公共利益的公务员队伍，调整得过且过、文过饰非、沾沾自喜的心态，工作向更高水准看齐，步伐向省级、国家级台阶迈进。鼓励创新、善待挫折，激励成功、宽容失误，营造干事创业、勇于创新的浓厚氛围。

　　建设优质服务型政府，做到“讲服务、有诚信、见百姓”。哪里有需要，就服务到哪里。用硬措施来整治软环境，加大问责追究力度。通过政务大厅、市长公开电话、政府网站以及信访接待等服务平台，及时公示人财物、规划、土地等审批程序及办理结果，与人民群众面对面，答民疑，解民忧。坚持诚信守约，创新服务方式，多研究怎么能行，少研究怎么不行，努力使四平成为环境优、成本低、回报高的北方创业城市。

　　建设效能管理型政府，做到“重管理、说行话、盯结果”。规范和理顺政府行政运行机制，决策民主快捷，执行果断有力。强化绩效考核，对政府各部门招商引资、项目建设、争取专项、总部经济、信访积案等工作情况，实行定期考评和向全社会公布，注重效果，鼓励先进，施压落后。把工作中心放到推动发展的重要任务上来，放到加快建设的重点工程上来，放到改善民生的重大课题上来，形成千军万马一条心、众人划桨开大船的良好氛围。

　　建设法治廉洁型政府，做到“用公权、守规矩、晒阳光”。坚持在市委的领导下开展工作，认真执行人大及其常委会决议，自觉接受人大法律监督、工作监督和政协民主监督，积极办理人大代表建议、政协委员提案。强化政府法制监督，加快推进阳光政务，完善政府新闻发布机制。全面落实党风廉政建设责任制，推进惩治和预防腐败体系建设，加强行政监察，加大政府投资、重大财政支出等项目审计监督力度，变事后审计为全程监督，树立廉洁、高效、务实、为民的政府形象。

　　各位代表！成就奠定基础，目标引领未来。让我们在市委的坚强领导下，全市上下团结一心，共图愿景，着力加快经济发展，突出改善民计民生，积极构建和谐社会，为早日把四平建设成为哈大经济大动脉上的一颗璀璨明珠，为使城乡居民生活得更加美好而努力奋斗！
